# Effect of intimate partner violence of women on minimum acceptable diet of children aged 6–23 months in Ethiopia: evidence from 2016 Ethiopian demographic and health survey

**DOI:** 10.1186/s40795-020-00354-7

**Published:** 2020-07-28

**Authors:** Desta Melaku Tsedal, Mezgebu Yitayal, Zegeye Abebe, Adino Tesfahun Tsegaye

**Affiliations:** 1 Department of Anesthesia, School of Medicine, University of Gondar, College of Medicine and Health Sciences, Gondar, Ethiopia; 2 Department of Health System and Policy, Institute of Public Health, University of Gondar, College of Medicine and Health Sciences, Gondar, Ethiopia; 3 Department of Human Nutrition, Institute of Public Health, University of Gondar, College of Medicine and Health Sciences, Gondar, Ethiopia; 4 Department of Epidemiology and Biostatistics, Institute of Public Health, University of Gondar, College of Medicine and Health Sciences, Gondar, Ethiopia

**Keywords:** C*hildren*, *Ethiopia*, *Intimate partner violence*, *Minimum acceptable diet*

## Abstract

**Background:**

The absence of proper infant and young child feeding practice results in malnutrition. Intimate Partner Violence (IPV) is potentially a major factor affecting child feeding practices. However, there is limited evidence about the effect of intimate partner violence (IPV) on a minimum acceptable diet. Therefore, in this study, we hypothesized that IPV will be associated with a lack of a minimum acceptable diet among children aged 6–23 months.

**Methods:**

We conducted a cross-sectional analysis using the Ethiopian Demographic and Health Survey (EDHS) 2016. All child-mother pairs that participated in EDHS 2016 from all regions of Ethiopia were included. The analysis included mother-child pairs where 6–23 months aged children with mothers who were ever in a committed partnership and interviewed for domestic violence were involved. The data were weighted considering enumeration areas as a cluster and place of residence as a stratum. A binary logistic regression analysis was done to identify factors independently associated with a minimum acceptable diet.

**Result:**

Totally, 1307 observations were included in the final analysis. The mean age of mothers was 29 years (standard deviation ±6.54 years), the mean age of children was 14. ± 5.02 months, and 32% of women had intimate partner violence (IPV). Of the children, 8% had a minimum acceptable diet (minimum acceptable diet), 15% had a minimum dietary diversity, and 43% had a minimum meal frequency. Having intimate partner violence decreases children minimum acceptable diet by 65% (AOR: **0.35**; 95% CI: **0.16, 0.77)**. The other factors associated with the minimum acceptable diet were caregivers attaining a secondary level of education (AOR: 4.01; 95% CI: 1.04, 15.45), currently working (AOR: 2.26; 95% CI: 1.01, 5.11), and undecided fertility desire (AOR: 4.72; 95% CI: 1.37, 16.28).

**Conclusion:**

Intimate partner violence against women had a negative association with the minimum acceptable diet children have received. Decreasing violence against women, educating, and increasing work opportunities for them would help in improving child feeding practice and reducing malnutrition and its consequences. Further studies that focus on possible community-based interventions aiming to decrease IPV are recommended.

## Background

For proper growth and development, children should receive the minimum acceptable diet including recommended quality and quantity of foods according to their age [1, 2]. Yet, over 150 million children are undernourished globally [3]. In Ethiopia, 38% of children under the age of 5 are short for their age, 10% are thin for their height, and 24% are thin for their age [4]. Most of the burden of childhood undernutrition can be explained by the absence of proper infant and young child feeding practices during the first two years [5, 6]. Child age, parity of mothers, child illness, maternal knowledge [7], the attitude of mothers [8], parental educational attainment, and household income are some of the factors that affect child feeding practices [9].

Intimate Partner Violence (IPV) is potentially a major factor affecting child feeding practices. IPV is a significant social and public health problem affecting 30% of ever-partnered women worldwide [10]. In Ethiopia, the cumulative incidence of IPV was 28% in a lifetime, and 20% in the past 12 months [4]. Women are the primary caregivers of children and violence affecting women might have an effect on child feeding practice. Violence is associated with mental health problems [11], and women who are depressed and anxious are more likely to abstain from breastfeeding and childcare [12]. Physical and psychological effects of IPV impair women’s ability to breastfeed and provide other complementary foods [13–17].

Previous studies focused on the effect of IPV on breastfeeding only and did not address the effect of IPV on the minimum acceptable diet. Continued investments in nutrition-specific interventions to avert child undernutrition through community engagement and women’s empowerment are recommended to accelerate progress in countries with the highest burden of child undernutrition and mortality [18]. Therefore, in this study, we hypothesized that maternal exposure to IPV will be associated with a lack of minimum acceptable diet among children aged 6–23 months, providing an indication that reduction in IPV may reduce childhood malnutrition.

## Methods

### Study setting and population

We conducted a cross-sectional analysis using the Ethiopian Demographic and Health Survey (EDHS) 2016. All child-mother pairs that participated in EDHS 2016 from all regions of Ethiopia were included. The analysis included mother-child pairs where 6–23 months aged children with mothers who were ever in a committed partnership and interviewed for domestic violence were involved.

### Sample size and sampling procedure

For EDHS 2016, the census frame was a complete list of 84,915 *enumeration areas* (EAs) created for the 2007 census. Each region was stratified into urban and rural areas and samples of EAs were selected in each stratum in two stages. In the first stage, a total of EAs were selected with probability proportional to EA size, and a household listing operation was performed in the selected EAs. The resulting lists of households served as a sampling frame for the selection of households in the second stage. Details of the recruitment of study participants found elsewhere [4]. From the involved households, 15,683 women completed the interview, and 5860 of them were interviewed for domestic violence. Only one eligible woman per household was randomly selected for domestic violence interview, and it was omitted if privacy could not be obtained. There were 10,641 children under the age of 5 and 3105 of them were in the age group between 6 and 23 months.

### **Data collection procedures** and quality control

The EDHS is a standardized report prepared from data collected using standardized tools. The data were collected through face-to-face interviews with the child’s mothers/caregivers. The 2016 EDHS questionnaires have parts divided into three; households, woman’s, and man’s questionnaires. Primary caregivers were interviewed to get data about the household and children. Generally, the questionnaires capture information on socio-demographic variables, reproductive health issues, domestic violence, health service characteristics, and others. The woman’s questionnaire had parts for children, which capture information on child immunization, health, and nutrition. The dietary diversity data were collected using a 24-h recall method; that is, mothers were asked to recall all foods given to their child in the past twenty-four hours before the survey.

### Variables of the study

The outcome of interest was getting minimum acceptable diet and the primary exposure was maternal exposure to intimate partner violence. The covariates considered were child characteristics (age in months, birth order, child sex); healthcare characteristics (place of delivery, number of antenatal visits, PNC counseling); parental characteristics (mother’s age, place of residence, father’s education, mother’s marital status, mother’s work status); household characteristics (wealth index, number of children under the age of 5, exposure to media).

**Minimum dietary diversity** (MDD) was defined as the proportion of children aged 6–23 months who received foods minimum acceptable diet from four or more food groups out of the seven food groups during the previous day/ within 24 h [9, 19]. The seven food groups are the following: starchy staples (foods minimum acceptable diet from grain, roots, or tubers); 2) legumes and nuts; 3) dairy products (milk other than breast milk, cheese, or yogurt); 4) Flesh foods (meat, fish, poultry and liver/organ meats; 5) vitamin A-rich fruits and vegetables (pumpkin; red or yellow yams or squash; carrots or red sweet potatoes; green leafy vegetables; fruits such as mango, papaya, or other local vitamin A-rich fruits); 6) other fruits and vegetables (or fruit juices); 7) eggs. **Minimum meal frequency (MMF)** was defined as [1] at ages 6–8 months, the child was breastfed and received two or more daily feedings of solid, semi-solid or soft foods; or [2] at ages 9 to 23 months, the child was breastfed and received three or more daily feedings of solid, semi-solid or soft foods; or [3] at ages 6 to 23 months, the child was not breastfed and received four or more daily feedings of solid, semi-solid, or soft foods. **Minimum acceptable diet:** A child was considered to receive at least the minimum acceptable diet for health if the MDD and the MMF criteria were met.

**Intimate partner violence** was defined as ever in committed partnership women who have experienced one or more of the specified acts of spousal physical violence or sexual violence or emotional violence by their current or most recent husbands/partners in the 12 months preceding the survey [20]. **Intimate partner physical violence** was defined as ever in committed partnership women who have experienced one or more of the specified acts of spousal physical violence by their current or most recent husbands/partners in the 12 months preceding the survey. Likewise, i**ntimate partner sexual violence and emotional violence were defined as ever** experiencing one or more of the specified acts of spousal sexual or emotional violence by their current or most recent husbands/partners in the 12 months preceding the survey.

**Exposure to mass media:** in the EDHS 2016 survey, respondents were asked how often they read a newspaper, listened to the radio, or watched television. Those who were exposed to any of the media *at least once a week* were considered to have adequate media exposure.

### Data management and analysis

The data were weighted considering enumeration areas as a cluster and place of residence as a stratum. Initial descriptive analyses provided general information on the characteristics of the study populations. A bivariate logistic regression analysis was done. Variables with *P*-value < 0.2 were included in the multivariable logistic regression model to identify factors independently associated with a minimum acceptable diet.

A propensity score matching analysis was performed to identify the effect of intimate partner violence on a minimum acceptable diet. A propensity score was the probability of being exposed to the IPV, given a set of observed covariates *(residence, mother educational status, wealth index, mother work status for the last 12 months, child desire, media exposure)*, and estimated using the logistic regression model. Nearest neighborhood matching was used in the analysis, which matches a given child of a woman who had intimate partner violence to a child of a woman who had no intimate partner violence whose propensity score is closest to that of the first subject or vice versa. The method is used to balance the two groups so that a direct comparison would be possible for evaluating the effects of intimate partner violence on the minimum acceptable diet. The average effect on children of women who had intimate partner violence was computed by averaging the difference between the outcomes of the two groups. The level of significance was defined at a *P*-value of less than 0.05.

## Results

### Sociodemographic and economic characteristics of the study participants

**Fig. 1:**
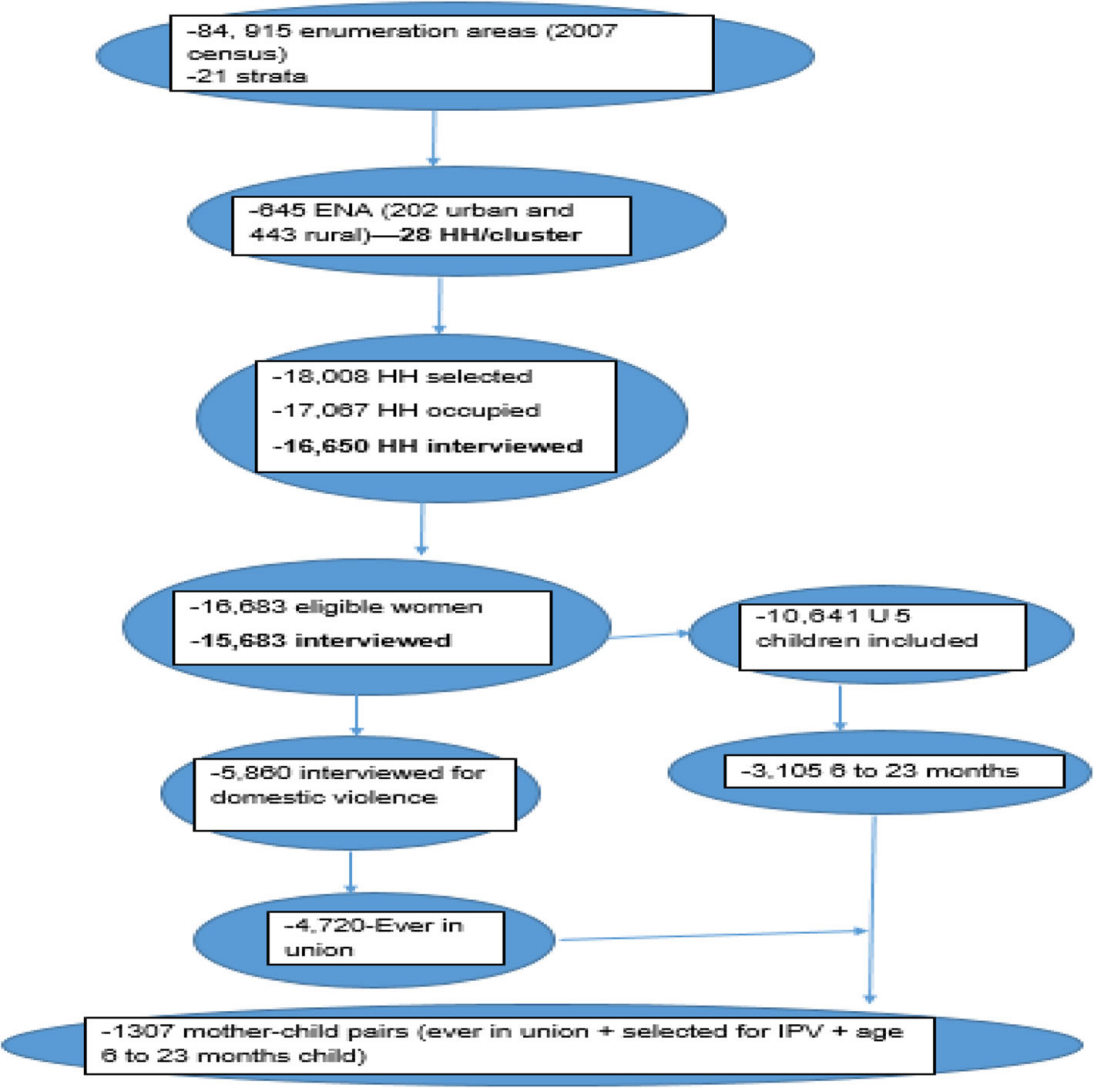
Flow chart showing the study procedure

**Fig. 2:**
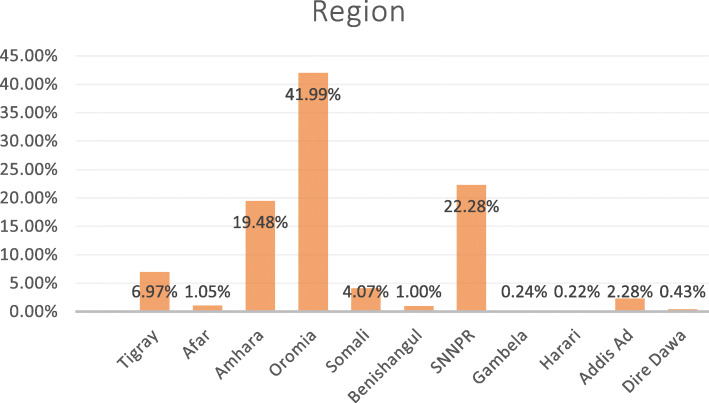
Distributions of study participants by regions, EDHS 2016

Of the total 15, 683 women participating, 5860 completed the questionnaire for domestic violence. Of those 5860, 1313 had children aged 6 to 23 months. Excluding six participants who were never in a committed partnership, 1307 observations were included in the final analysis (Fig. 1). The mean age of mothers was 29 years (standard deviation ±6.54 years), 30% of the mothers were in the age group between 25 and 29 years, 88% were from rural areas, 62% had no formal education and 23% were in the 1st wealth quantile (poorest). Also, 47% of the study participants’ partners had no formal education (Table 1). Regarding regional composition, 42% of the study participants were from the Oromia region and only 0.22% were from Hariri region (Fig. 2).

**Table 1:** Sociodemographic and economic characteristics of parents of children aged 6 to 23 months in Ethiopia, EDHS 2016

Variables	Intimate partner violence		Total
Maternal age	Yes N (%)	No N (%)	N (%)
15–19	5 (1)	44 (5)	49 (4)
20–24	90 (21)	188 (21)	278 (21)
25–29	119 (28)	272 (31)	392 (30)
30–34	101 (24)	196 (22)	297 (23)
35–39	72 (17)	128 (14)	199 (15)
40–44	27 (6)	49 (6)	76 (6)
45–49	7 (2)	9(1)	16 (1)
**Residence**			
Urban	33 (8)	118 (13)	151 (11)
Rural	388 (92)	768 (87)	1156 (88)
**Maternal education**			
No education	271 (64)	539 (61)	810 (62)
Primary	120 (29)	260 (29)	380 (29)
Secondary	19 (4)	56 (6)	75 (6)
Higher	11 (3)	31 (4)	42 (3)
**Religion**			
Orthodox	140 (33)	323 (36)	463 (35)
Protestant	96 (23)	192 (22)	289 (22)
Muslim	164 (39)	355 (40)	519 (40)
Others	20 (5)	16 (2)	36 (3)
**Household Wealth Index**			
Poorest	119 (28)	183 (21)	302 (23)
Poorer	94 (22)	196 (22)	289 (22)
Middle	95 (23)	185 (21)	280 (21)
Richer	71 (17)	175 (20)	246 (19)
Richest	42 (10)	147 (17)	189 (14)
**Father’s educational status**			
No education	201 (48)	417 (47)	618 (47)
Primary	189 (45)	330 (37)	519 (40)
Secondary	23 (6)	89 (10)	112 (9)
Tertiary	8 (2)	50 (6)	58 (4)

### Reproductive characteristics of mothers/caregivers

For women who had given birth, the mean parity was 4.10 (SD ± 2.52) births. The age at marriage of 36% of the women were below 18 years, 56% of the women had more than one child under the age of 5 years, and 6% of the women were pregnant during the survey (Table 2).

**Table 2:** Reproductive characteristics of mothers/caregivers of children aged 6 to 23 months in Ethiopia, EDHS 2016

Variables	Intimate partner violence		Total
Children ever born	Yes N (%)	No N (%)	N (%)
1 to 2	126 (30)	318 (36)	444 (34)
3 to 4	93 (22)	256 (29)	348 (27)
> = 5	202 (48)	313 (35)	515 (39)
**Presence of other under five children in the house**			
No	163 (39)	407 (46)	570 (44)
Yes	258 (61)	479 (54)	737 (56)
**Age at first birth**			
< 18	150 (36)	315 (36)	466 (36)
18–24	244 (58)	492 (56)	736 (56)
25–29	24 (6)	63 (7)	87 (7)
> =30	3 (1)	15 (2)	18 (1)
**Currently pregnant**			
No or unknown	394 (94)	837 (95)	1231 (94)
Yes	27 (6)	49 (5)	76 (6)
**Number of living children**			
< =2	134 (32)	347 (39)	481 (37)
3 to 4	116 (27)	281 (32)	396 (30)
> =5	171 (41)	258 (29)	429 (33)
**Current Marital Status**			
Married	396 (94)	855 (96)	1151 (96)
Living with partner	7 (2)	15 (2)	22 (2)
Others^a^	18 (4)	17 (2)	35 (2)
**Fertility desire**			
Want more children	249 (59)	516 (58)	765 (59)
Undecided	26 (6)	41 (5)	67 (5)
Don’t want more children	146 (35)	329 (37)	475 (36)
**Mother worked outside home in the last 12 months**			
No	227 (54)	506 (57)	733(56)
In the past year	77 (18)	157 (18)	234 (18)
Currently working	118 (28)	223 (25)	340 [21]

^a^Widowed, divorced, and separated

### Media exposure

**Fig. 3:**
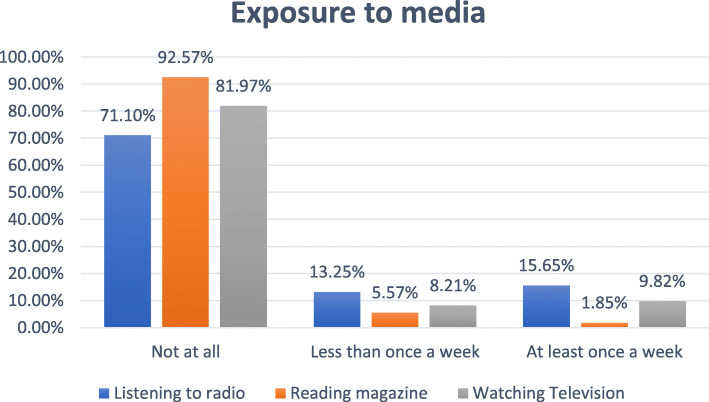
Media exposure status of care givers of children aged between 6 to 23 months in Ethiopia, EDHS 2016. *Experiencing either of the three violence types

Almost one-fifth (20%) of the participants had satisfactory media exposure. About 16% of the participants had listened to the radio at least once a week (Fig. 3).

### Child-related characteristics

The mean age of children was 14.15 ± 5.02 months with 37% between 12 and 17 months, 53% were girls, 29% were 6th and above in terms of their birth order (Table 3).

**Table 3:** Characteristics of children aged 6 to 23 months in Ethiopia, EDHS 2016

Variables	Intimate partner violence		Total
Birth order	Yes N (%)	No N (%)	N (%)
First	70 (17)	160 (18)	230 (18)
Second	56 (13)	160 (18)	216 (17)
Third	49 (12)	144 (16)	192 (15)
Fourth	50 (12)	115 (13)	164 (13)
Fifth	44 (10)	88 (10)	131 (10)
6th and above	153 (36)	221 (25)	374 (29)
**Twin**			
No	411 (98)	843 (95)	1254 (96)
Yes	10 (2)	43 (5)	53 (4)
**Sex of child**			
Male	213 (51)	403 (46)	616 (47)
Female	208 (49)	483 (54)	691 (53)
**Birth interval**			
First birth	70 (17)	164 (19)	234 (18)
Less than two years	66 (16)	139 (16)	205 (16)
2 to 5 years	232 (55)	441 (50)	674 (52)
More than 5 years	53 (13)	141 (16)	194 (15)
**Child age in months**			
6 to 11 months	120 (28)	313 (35)	433 (33)
12–17 months	167 (40)	319 (36)	486 (37)
18–23 months	134 (32)	254 (29)	388 (30)
**Mother had ANC during pregnancy of the child**			
No	167 (40)	334 (38)	501 (38)
Yes	254 (60)	552 (62)	806 (62)
**Duration of breastfeeding**			
Ever breast fed	63 (15)	134 (15)	197 (15)
Never breast fed	7 (2)	42 (5)	49 (4)
Still breast feed	351 (83)	710 (80)	1061 (81)
**Wanted status of child during pregnancy**			
Wanted during pregnancy	295 (70)	676 (76)	970 (74)
Wanted later	81 (19)	137 (15)	218 (17)
Not wanted	45 (11)	73 (8)	118 (9)
**Child delivered in a health facility**			
No	292 (69)	541 (61)	833 (64)
Yes	129 (31)	345 (39)	474 (36)
**Size at birth**			
Above average	154 (37)	277 (31)	430 (33)
Average	153 (36)	383 (43)	536 (41)
Below average	114 (27)	227 (25.58)	341 (26.07)
**Index to birth history**			
Last birth	402 (96)	845 (95)	1247 (95)
Not last birth	19 (4)	41 (5)	60 (5)

### Intimate partner violence

**Fig. 4:**
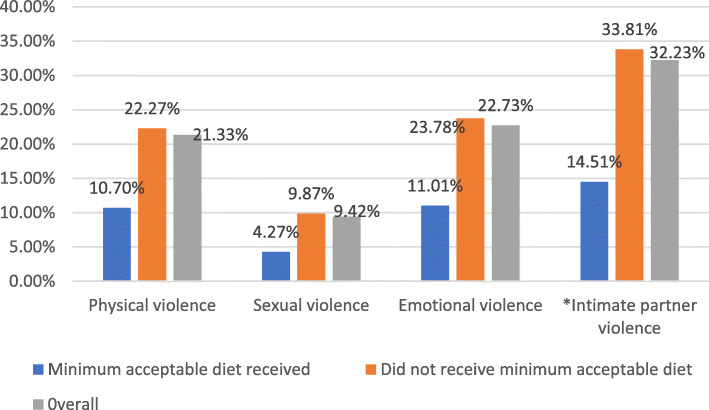
*Experiencing either of the three violence types. Intimate partner violence among care givers of children aged between 6 to 23 months in Ethiopia, EDHS 2016

Overall, 32% of the study participants experienced IPV with emotional violence as the most common type of IPV (Fig. 4).

### Feeding practice of children

In this study, 8% of children received the recommended minimum acceptable diet. Among the children, 42% were given the recommended meal frequency, and 14% received the recommended minimum dietary diversity.

### Determinants of minimum acceptable diet

In the multivariable analysis, level of education, mothers’ work status, fertility desire, and intimate partner violence had a statistically significant association with a minimum acceptable diet. The odds of getting a minimum acceptable diet among children of mothers who had a secondary education was higher than those who did not have formal education (AOR: **4.01**; 95% CI: **1.04, 15.45**). As compared to children of mothers who did not work for the last 12 months prior to the survey, children of mothers who were working during the survey had higher odds of getting a minimum acceptable diet (AOR: **2.26**; 95% CI: **1.01, 5.11)**. Children of mothers who didn’t decide their fertility desire had higher odds of getting a minimum acceptable diet as compared to those who were children of mothers who decided not to have more children (AOR: **4.72;** 95% CI: **1.37, 16.28**). Finally, children of mothers who had intimate partner violence had 65% lesser odds of getting a minimum acceptable diet as compared to those who were children of women who did not have an intimate partner violence (AOR: **0.35**; 95% CI: **0.16, 0.77)** (Table 4).

**Table 4:** Factors associated with minimum acceptable diet among children aged 6 and 23 months in Ethiopia, EDHS 2016

Variables	Minimum acceptable diet			AOR (95% CI)
	Yes N (%)	No N (%)	COR (95% CI)	
**Intimate partner violence**				
Yes	16 (4)	406 (96)	**0.33 (0.15, 0.74)**	**0.35 (0.16, 0.77)**
No	91 (10)	794 (90)	1.00	1.00
**Residence**				
Urban	33 (22)	118 (78)	1	1
Rural	74 (6)	1082 (94)	0.24(0.11, 0.57)	0.74(0.14, 3.87)
**Maternal Education**				
No formal education	51 (6)	758 (94)	1.00	1
Primary education	23 (6)	357 (94)	0.96 (0.46, 2.02)	0.89 (0.43, 1.85)
Secondary education	19 (25)	56 (75)	**4.85 (1.33, 17.71)**	**4.01 (1.04, 15.45)**
Higher education	14 (32)	29 (68)	**6.92 (1.85, 25.87)**	3.44 (0.44, 26.72)
Wealth Index^*^				
Poorest	17 (6)	286 (94)	1.00	1
Poorer	19 (7)	270 (93)	1.23(0.42, 3.59)	0.97 (0.33, 2.82)
Middle	20 (7)	260 (93)	1.30(0.41, 4.10)	1.15 (0.38, 3.52)
Richer	16 (7)	230 (93)	1.20(0.34, 4.30)	0.67 (0.18, 2.47)
Richest	35 (18)	154 (82)	**3.87(1.40, 10.73)**	0.68 (0.11, 4.18)
**Presence of other under five children in the house**				
Yes	48 (7)	689 (93)	0.60 (0.28, 1.31)	0.73 (0.31, 1.72)
No	59 (10)	511 (90)	1	1
**Currently Breastfeeding**				
Yes	97 (9)	964 (91)	3.49 (0.68, 17.92)	4.36 (0.69, 27.56)
No	7 (3)	239 (97)	1.00	1
**Fertility Desire**				
Want More children	65 (8)	701 (92)	1.43 (0.59, 3.48)	1.12 (0.45, 2.77)
Undecided	13 (20)	54 (80)	**3.81 (1.20, 12.14)**	**4.72 (1.37, 16.28)**
Don’t Want any	29 (6)	446 (94)	1.00	1.00
**Mother worked outside home in the last 12 months**				
No	43 (6)	690 (94)	1.00	1
Worked in 12 months	16 (7)	218 (93)	1.25 (0.35, 3.82)	1.25 (0.40, 3.91)
Currently working	48 (14)	292 (86)	**2.68 (1.21, 5.90)**	**2.26 (1.01, 5.11)**
**Child delivered in a health facility**				
Yes	53 (11)	421 (89)	1.80(0.89, 3.66)	0.79 (0.39, 1.60)
No	54 (6)	779 (94)	1.00	1.00
**Media Exposure**				
Adequate	44 (17)	211 (83)	**3.32 (1.65, 6.65)**	2.29 (0.92, 5.67)
In Adequate	63 (6)	989 (94)	1.00	1.00

## Discussion

This study aimed to assess the effect of maternal/caregiver IPV exposure on getting the minimum acceptable diet among children aged 6 to 23 months. We found that maternal exposure to IPV was associated with a lower proportion of children receiving a minimum acceptable diet. Also, higher maternal level of education, mother/caregivers’ currently working, and desire for children had a statistically significant association with getting a minimum acceptable diet.

We found a large effect, with the proportion of children receiving a minimum acceptable diet declining by 65% among families in which women experienced IPV. This implies that the effect of IPV exposure to mothers/caregivers has the potential to pass to their children and lead them to develop malnutrition and related consequences. Knowing IPV as it has a compounded effect in affecting children’s growth and development beyond its consequence on women helps to broaden perspectives in implementing nutrition-related programs. This finding is consistent with studies done in India [22], Bangladesh [23], and a systematic review that included many countries [24]. When women encounter violence from their partners, the care they provide for their children decreases. When violence happens, it has a persistent negative psychological effect on the victims [16, 24, 25], weakening the mother-child bond [21, 26] and making women less autonomous [11, 27]. When caregivers mind is occupied by negative thoughts that could be associated with some mental health problems, and their productivity and care to their children decrease. Women who faced intimate partner violence are more likely to abandon their children; this would prevent the child to get the appropriate care and nutrition he/she needs [24]. Finally, this would end up with malnutrition in the children and socioeconomic derangement to the society at large [23, 28, 29].

Children of women who had secondary education were more likely to get a minimum acceptable diet than those who had no formal education. This is supported by studies done in Sri Lanka [30], Nepal [31], and Ethiopia [32]. Educated women are closer to media and have better awareness about child feeding practices and they could provide an appropriate diet for their children [33]. Most of the time, educated people live in urban areas where they could have better food and health care access with more work opportunities to support their families [34, 35].

Children of mothers who had a job outside of their home and were working during the survey were more likely to get a minimum acceptable diet than those who were not working. Having income helps women to have better participation in budgeting household expenditures in a way that positively influences [36] the variety and frequency of diet they provide for their children [30, 37]. Moreover, this group of women could be more autonomous and educated and could make better decisions about household food consumption [32].

As compared to those who did not want to have more children, children of women who did not decide their future fertility were more likely to get a minimum acceptable diet. Women who did not decide could be in the dilemma of having more children or not. This feeling might happen due to the affection of their current children that possibly followed by better care and the provision of diversified and frequent meals.

Although this study considered nationwide data, all participating women were not interviewed for IPV, and information about IPV might not represent the national burden. Women were asked to recall an IPV happen within 12 months and there could be a tendency not to recall incidents and that would lead to underestimation of IPV. Also, recalling the food children fed would not be easy and cause misclassification of feeding practice, though it was minimized by using only a 24 h recall period. In addition, women could be shy to report as they encountered violence and that would lead to social desirability bias and underestimate the burden of IPV and thereby its effect on a minimum acceptable diet. Due to the cross-sectional nature of the data, temporality is still a concern. Although the status of getting a minimum acceptable diet was a more incident event and assessed using a 24 h recall period, a reverse temporal relationship could not be precluded. Future researchers would bring more valid evidence and further understand the mechanism by conducting prospective studies that take into account the sensitiveness of the issue and possible mechanisms to handle it. Besides, implementations of nutritional programs need to take maternal partner violence into consideration. Addressing maternal situations could have its own impact on the strategies of halting childhood malnutrition.

## Conclusion

Overall, intimate partner violence against women had a negative association with the minimum acceptable diet children have received. Decreasing violence against women, educating, and increasing work opportunities for them would help in improving child feeding practice and reducing malnutrition and its consequences. Further studies that focus on possible community-based interventions aiming to decrease IPV are strongly recommended.

## Data Availability

The datasets used and/or analyzed during the current study are available from the corresponding author on reasonable request**.**

## Abbreviations

AOR: Adjusted Odds Ratio
BMI: Body Mass Index
COR: Crude Odds Ratio
CSA: Central Statistical Agency
DD: Dietary Diversity
EDHS: Ethiopian Demographic and Health Survey
IPV: Intimate Partner Violence
IYCF: Infant and Young Child Feeding
MDD: Minimum Dietary Diversity
MMF: Minimum Meal Frequency
OR: Odds Ratio
SD: Standard Deviation
WHO: World Health Organization
